# Nutritional Status and Physical Activity Levels in Adult Patients with Phenylketonuria

**DOI:** 10.3390/nu18111804

**Published:** 2026-06-03

**Authors:** Damla Kalkan, Yılmaz Yıldız, Yiğitcan Karanfil, Feza Korkusuz, Ali Dursun, Serap Sivri, Hülya Gökmen Özel

**Affiliations:** 1 Nutrition and Dietetic Department, Hacettepe University İhsan Doğramacı Children’s Hospital, 06100 Ankara, Türkiye; damla.yildirim@hacettepe.edu.tr; 2 Division of Metabolism, Department of Pediatrics, Faculty of Medicine, Hacettepe University, 06100 Ankara, Türkiye; yilmaz.yildiz@hacettepe.edu.tr (Y.Y.);; 3 Department of Sports Medicine, Faculty of Medicine, Hacettepe University, 06100 Ankara, Türkiye; yigitcan.karanfil@hacettepe.edu.tr (Y.K.);; 4 Department of Nutrition and Dietetics, Faculty of Health Sciences, Hacettepe University, 06100 Ankara, Türkiye

**Keywords:** phenylketonuria, classical PKU, phenylalanine restriction, medical nutrition therapy, exercise behavior, physical activity

## Abstract

**Background/Objectives:** Phenylketonuria (PKU) is a rare autosomal recessive disorder caused by phenylalanine hydroxylase (PAH) deficiency, impairing the conversion of phenylalanine (Phe) to tyrosine. Although early diagnosis and intervention yield excellent outcomes, dietary adherence often declines in adulthood, potentially leading to poor metabolic control and adverse nutritional consequences. This study aimed to evaluate physical activity levels, nutritional status, metabolic control, and anthropometric outcomes in adults with classic PKU, which have not been sufficiently researched in the current literature. **Methods:** This cross-sectional study included 100 adults with classical PKU (cPKU; baseline phenylalanine levels ≥ 1200 µmol/L) under regular follow-up at the Division of Metabolism, Hacettepe İhsan Doğramacı Childrens’ Hospital. Sociodemographic traits and dietary behaviors were evaluated through structured interviews carried out by a dietitian. Dietary intake was assessed by using a 24 h dietary recall method, and nutrient analyses were performed with the Bebis 7.2 software program. Using the short version of the International Physical Activity Questionnaire (IPAQ), physical activity levels were specified, and participants were categorized according to established scoring criteria. **Results:** A hundred adults with classical PKU took part in the study, including 47 males and 53 females, with a mean age of 23.84 ± 5.41 years; 5% of participants were underweight, 40% had normal weight, 39% were overweight, and 16% were listed as obese. The intake of mean daily energy is 2443.8 ± 384.6 kcal for men and 1822.5 ± 312.7 kcal for women. Carbohydrates contributed approximately 61% of total daily energy intake in both genders, whereas protein accounted for 12–13% and fat for approximately 26–27% of total energy intake; 17% of participants were physically inactive, 40% were minimally active, and 43% met criteria for sufficient physical activity according to IPAQ-based classification. Energy intake, the use of Phe-free protein substitutes, and BMI were significantly higher in the sufficiently active group compared to the low-active group in men, while no significant differences were observed between physical activity groups among women. **Conclusions:** Adults with classical PKU showed a high prevalence of overweight and obesity, together with differences in dietary intake and physical activity patterns. Physical activity levels were associated with several nutritional and metabolic characteristics; however, further long-term research is required to fully understand these connections.

## 1. Introduction

Phenylketonuria (PKU) is an autosomal recessive disorder caused by pathogenic variants in the phenylalanine hydroxylase (PAH) gene, which encodes PAH, the hepatic enzyme essential for converting phenylalanine (Phe) to tyrosine [1,2]. PAH deficiency leads to hyperphenylalaninemia, with untreated blood Phe levels often exceeding 1200 μmol/L in classical PKU [2], resulting in severe intellectual disability and irreversible brain damage if not managed promptly [1]. Even with early treatment, elevated Phe levels are inversely associated with neurological outcomes, including deficits in executive function, processing speed, motor skills, cognition, and intelligence quotient (IQ) [3].

Lifelong nutritional therapy that focuses on limiting Phe is the most important part of treating PKU. In childhood, treatment goals prioritize optimal growth and neurodevelopment, whereas adulthood emphasizes preservation of neurocognitive stability and psychological well-being. To control Phe intake, PKU dietary management requires restriction on natural protein, supplementation with Phe-free L-amino acid mixtures to meet protein requirements, and consumption of low-protein foods to maintain adequate energy balance. Guidelines recommend maintaining blood Phe at 120–360 µmol/L across all ages [4], with lifelong treatment to avert neurological risks [5,6]. While target concentrations of 120–360 µmol/L are frequently reported, recent European guidelines for adults with PKU accept blood Phe concentrations ≤600 µmol/L as an appropriate threshold for metabolic control in adulthood. Although dietary Phe restriction appears theoretically straightforward [7], sustaining long-term adherence to a low-Phe diet poses considerable psychosocial and practical barriers, particularly during adolescence, early adulthood, and pregnancy [1,8,9,10,11].

As treatment outcomes and quality of life improve, adult people with PKU are taking on more social roles, such as getting married, having children, traveling, and doing regular physical activity or sports [12]. This broader engagement in daily life may further challenge adherence to the stringent low-phenylalanine diet essential for metabolic control. The latest European guidelines on PKU feature a dedicated section on physical exercise, highlighting its critical role in preserving metabolic control among active individuals. These recommendations emphasize strategic timing and dosing of amino acid mixtures, alongside targeted carbohydrate and fat consumption, coupled with adequate hydration, to safeguard blood Phe homeostasis while enhancing exercise performance and recovery [10].

In Türkiye, the first newborn screening program started as a pilot program in maternity hospitals of the 27 cities’ metropolitan areas in 1986 and has been implemented nationwide since 2006 [13,14]. Since then, the number of adult PKU patients has been increasing.

Considering that an individual’s physical activity level is an integral part of evaluating their nutritional status, which affects body composition, energy expenditure, and nutrient requirements, its assessment may provide clinically relevant information in adults with PKU. Additionally, it can facilitate metabolic control and a more detailed examination of disease management in adults with PKU, leading to improved health outcomes and better adherence to dietary recommendations. Moreover, as physical activity levels may influence energy intake, protein requirements, protein substitute use, and consequently phenylalanine intake, dietary monitoring is particularly important in this population. This study aimed to evaluate the nutritional status and physical activity levels of PKU patients aged ≥18 years.

## 2. Subjects and Methods

### 2.1. Subjects

One hundred adult patients diagnosed with classical phenylketonuria (cPKU), defined as baseline phenylalanine (Phe) concentrations ≥ 1200 µmol/L, from the Division of Pediatric Metabolism, Hacettepe İhsan Doğramacı Children’s Hospital, were recruited. Of the 100 subjects, 47 were male and 53 were female, with a mean age of 23.8 ± 5.4 years (interquartile range 20–27 years); exclusion criteria included (i) severe intellectual disability, (ii) the presence of concomitant musculoskeletal, endocrine, or cardiovascular disorders, (iii) current pregnancy or planned pregnancy during the study period, (iv) treatment with large neutral amino acids (LNAA) or tetrahydrobiopterin (BH4), and (v) age below 18 years. Between October 2025 and January 2026, all adult patients with classical PKU who were followed at the Metabolism Unit, met the inclusion criteria, and agreed to participate were included in the study.

This study was approved by the Clinical Research Ethics Committee of Hacettepe University (Decision No: 2025/17-12, KA-23015, approval date 16 September 2025). Written informed consent was obtained from the patients.

### 2.2. Study Design

In this single-center cross-sectional study, participants were interviewed to obtain sociodemographic, clinical background, and dietary information by a trained dietitian. Nutritional intake was assessed using the 24 h dietary recall method; physical activity levels were evaluated based on self-reported activity patterns. In addition, anthropometric measurements together with plasma Phe concentrations were assessed. Clinical characteristics including age at diagnosis, treatment status, plasma phenylalanine concentrations, and metabolic control status were obtained from medical records. 

### 2.3. Assessment of Plasma Phenylalanine Concentrations

Plasma phenylalanine (Phe) concentration at the time of diagnosis and the latest plasma Phe concentration measured during routine follow-up visits were evaluated. Plasma Phe concentrations were assessed according to the European guidelines for adults [9]. Plasma phenylalanine concentrations were measured in the Hacettepe University Metabolic Laboratory using high-performance liquid chromatography (HPLC) with a Shimadzu Prominence system (Shimadzu Corporation, Kyoto, Japan) according to standard laboratory procedures. The median blood Phe concentration for the previous 3-year period was used as an indicator of metabolic control. Individuals with plasma Phe concentrations of 600 µmol/L and below were classified as having adequate metabolic control according to recent European guidelines for adult patients with PKU, while those with levels above were classified as having inadequate metabolic control [10].

### 2.4. Classification of Diagnosis Age

Age at diagnosis was categorized in line with formerly established criteria [10]. Patients diagnosed between three months and seven years of age were classified as late-diagnosed, and those diagnosed within the first three months of life were categorized as early-diagnosed.

### 2.5. Assessment of Anthropometric Measurements

Using a calibrated digital scale and wall-mounted stadiometer (Seca, Hamburg, Germany), body weight and height were measured in the morning under fasting conditions at the diet outpatient clinic. Body mass index (BMI) was calculated as body weight in kilograms divided by height in meters squared (kg/m^2^) and classified according to the World Health Organization (WHO) criteria [15].

### 2.6. Assessment of Dietary Intake

Dietary intake data were obtained by using a single 24 h dietary recall. Participants were asked to report all foods and beverages consumed during the previous day. Sizes of portions were estimated using a photographic food atlas to enhance accuracy [16].

Analysis of nutrients was performed by using the Nutrition Information Systems software (BEBIS, version 7.2, Nutrition Information Systems İstanbul, Türkiye) [17]. Total daily energy intake (kcal/day); total protein intake (g/day and percentage of total energy); natural protein intake (g/day and percentage of total protein); protein derived from phenylalanine-free amino acid substitutes (g/day and percentage of total protein); carbohydrate intake (g/day and percentage of total energy); fat intake (g/day and percentage of total energy); and daily dietary phenylalanine intake (mg/day) were calculated.

### 2.7. Assessment of Physical Activity

Physical activity levels were assessed using the International Physical Activity Questionnaire (IPAQ). Validity and reliability have been established by the Turkish version of the instrument [18], and official permission was obtained.

Seven items of assessing time (minutes) and frequency (days) spent during the previous seven days in sitting, walking, moderate-intensity activities (e.g., carrying light loads, cycling at a regular pace, traditional dancing, bowling, or doubles tennis), and vigorous-intensity activities (e.g., heavy lifting, digging, aerobics, basketball, football, or fast cycling) were involved in the questionnaire.

Physical activity was quantified in metabolic equivalents of task (MET) units, where MET corresponds to the ratio of a given activity’s energy cost to its resting metabolic rate. In other words, 1 MET approximates energy expenditure at rest. For each activity category, duration (minutes) and frequency (days) were multiplied by standardized MET coefficients (sitting: 1.5 METs; walking: 3.3 METs; moderate activity: 4.0 METs; vigorous activity: 8.0 METs) to calculate MET-minutes per week. Total MET-min/week values were subsequently classified according to established IPAQ scoring protocols [19].

If participants engaged in vigorous-intensity activity on at least three days per week, accumulating ≥1500 MET-min/week, or if they achieved ≥3000 MET-min/week through any combination of walking, moderate, or vigorous activities across seven days, they were considered physically active.

A moderate (minimally active) level was defined as the following:

Vigorous activity for at least 20 min per day on three or more days per week;

Moderate-intensity activity and/or walking for at least 30 min per day on five or more days per week; or

Any combination of walking, moderate, or vigorous activities on three to five or more days, accomplishing ≥600 MET-min/week.

Individuals that did not meet the criteria for either moderate or high activity levels were listed as physically inactive [19]. Total energy expenditure relevant to physical activity was calculated based on individual MET scores [19].

### 2.8. Statistical Analysis

All statistical analyses were performed using SPSS software (version 20.0, IBM Corp., Armonk, NY, USA). Continuous variables were presented as mean ± standard deviation (SD) when normally distributed and as median with interquartile range (Q1–Q3) when normality assumptions were not met. Categorical variables were expressed as percentages.

Using both the Kolmogorov–Smirnov and Shapiro–Wilk tests, the distribution of continuous variables was evaluated. The Independent Samples *t*-test was used for comparisons between two independent groups when parametric assumptions were met, while variables that did not satisfy normality assumptions were logarithmically transformed (log10) prior to analysis. Spearman correlation analysis was performed to evaluate associations between physical activity parameters, anthropometric measurements, last measured plasma phenylalanine concentration, and dietary variables. Multivariable linear regression analysis was conducted to identify independent variables associated with last measured plasma phenylalanine concentration after adjustment for potential confounding variables, including age, sex, BMI, energy intake, protein equivalent intake from Phe-ree protein substitutes, and physical activity level. A *p*-value of less than 0.05 was regarded as statistically significant.

## 3. Results

### 3.1. Plasma Phenylalanine Concentrations and Age at Diagnosis

The mean plasma Phe concentration at diagnosis was 1698 ± 582 µmol/L. The mean last measured plasma Phe concentration was 834 ± 438 µmol/L, and 65% of patients had Phe concentrations above the recommended target range for adults according to the European guidelines (Table 1).

The median age at diagnosis was 30 days (interquartile range 14 days to 58.5 days); ten children were diagnosed after 3 months of age (median age 6 months; range 127 days to 1 year).

### 3.2. Anthropometry

Mean BMI values seemed to be lower in males (25.9 ± 4.5 kg/m^2^) compared to females (27.3 ± 5.3 kg/m^2^), but the difference was not statistically significant (*p* > 0.05). Five percent of the participants were underweight, 40% had normal weight, 39% were overweight, and 16% were classified as obese according to WHO BMI classification. A higher number of males were within the normal weight category compared to females (40.4% vs. 39.6%) and the prevalence of overweight was higher in males, whereas obesity prevalence was higher in females (Table 2).

### 3.3. Dietary Intake

Despite the doctor’s recommendation, four patients were not taking Phe-free protein substitutes (1 male, 3 females). Thirty-four patients (16 males, 18 females) took a protein substitute once or twice a day despite recommendations to take them in more frequent doses.

Mean daily energy intake was 2443.8 ± 384.6 kcal in males and 1822.5 ± 312.7 kcal in females.

The percentage of total energy derived from carbohydrates was 60.9 ± 7.4% in males and 60.5 ± 6.6% in females; from protein, 12.2 ± 2.6% and 13.0 ± 3.2%; and from fat, 26.8 ± 7.4% and 26.4 ± 7.2%, respectively. In terms of dietary fat or protein percentage in both genders (*p* > 0.05), no significant differences were observed between those with adequate vs. inadequate metabolic control.

Mean daily protein intake was 1.0 ± 0.2 g/kg in males and 0.9 ± 0.2 g/kg in females, and there was no statistically significant difference between patients with adequate vs. inadequate metabolic control. Yet, carbohydrate contribution to total energy intake was remarkably higher in males with blood Phe ≤ 600 µmol/L compared to those with levels > 600 µmol/L (64.1 ± 8.9% vs. 59.3 ± 6.0%, *p* < 0.05).

Mean protein intake from protein substitutes seemed to be higher among females with blood Phe ≤ 600 µmol/L compared with those with blood Phe > 600 µmol/L (71.9 ± 14.5% vs. 66.6 ± 10.6%), but this difference was not statistically significant (*p* = 0.165). Similarly, in both genders, mean protein intake from protein substitutes tended to be higher among individuals with blood Phe ≤ 600 µmol/L compared to those with levels > 600 µmol/L, but these differences were not statistically significant (males *p* = 0.064; females *p* = 0.165).

Mean daily dietary phenylalanine intake was 1218.9 ± 612.5 mg in males and 936.2 ± 683.4 mg in females. Within each gender, individuals with blood Phe ≤ 600 µmol/L had lower dietary phenylalanine intake compared to those with >600 µmol/L (males: 1150.7 ± 776.9 vs. 1254.1 ± 519.3 mg/day, *p* > 0.05; females: 829.9 ± 611.4 vs. 1074.9 ± 758.8 mg/day, *p* > 0.05); however, these differences were not statistically significant (Table 3).

### 3.4. Physical Activity

Median total physical activity was 3576 MET-min/week (1848–5820) in males and 1230 MET-min/week (396–1980) in females according to IPAQ scores. Based on IPAQ classification, 2.1% of males were physically inactive, 27.7% were minimally active, and 70.2% achieved sufficient activity levels. In contrast, 50.9% of females were minimally active, and only 18.9% met criteria for sufficient physical activity (Table 4).

Among males categorized as sufficiently active (70.2%), 63% reported engaging in structured exercise, while 37% were employed in physically demanding occupations (e.g., fishing, factory work, load carrying). Among sufficiently active females (18.9%), 60% performed regular exercise, and 40% were involved in labor-intensive work (e.g., agricultural activities).

Forty-five percent of the total sample reported engaging in vigorous-intensity physical activity; of these, 76% were males and 24% were females. Moderate-intensity activity was reported by 67% of participants (64% males, 36% females); 420 min per week was the median sitting time.

It was observed that sufficiently physically active men had higher energy and Phe-free protein substitute intake compared with the low-activity group (2565 ± 381 vs. 2158 ± 201.3 kcal/day and 47.2 ± 9.7 vs. 39.1 ± 8.5 g/day, respectively), and this difference is statistically significant (*p* < 0.0001, *p* = 0.008). At the same time, BMI values were also found to be higher in the adequately active group (26.9 ± 4.4 vs. 23.7 ± 4.1 kg/m^2^), and this difference is statistically significant (*p* = 0.025). In women, the sufficiently active group had higher energy (1996.6 ± 436.1 vs. 1782 ± 267.2 kcal/day), protein (58.9 ± 22.1 vs. 56.1 ± 12.0 g/day), and Phe-free protein substitute intake (44.6 ± 26.3 vs. 39.1 ± 10.8 g/day); however, these differences were not statistically significant (*p* = 0.067, *p* = 0.787, *p* = 0.672, respectively). Similarly, the BMI value was higher in sufficiently active women (26.6 ± 5.5 vs. 24.8 ± 5.3 kg/m^2^), whereas the last measured plasma Phe level was lower in adequately active women (618 ± 336 vs. 732 ± 384 µmol/L), but no difference was statistically significant (Table 5).

Sixteen percent of overweight and obese individuals were inactive, and 31% were minimally active when physical activity levels were analyzed according to BMI categories (Table 6).

In males, a significant moderate positive correlation was found between IPAQ score and energy intake (r = 0.443, *p* = 0.002) and between plasma Phe concentration and BMI (r = 0.434, *p* = 0.002). In females, BMI had weak positive correlation with plasma Phe concentration (r = 0.276, *p* = 0.045), protein intake (r = 0.338, *p* = 0.013), and protein equivalent intake from Phe-free protein substitutes (r = 0.292, *p* = 0.044). Moreover, protein intake had a weak positive correlation with energy intake (r = 0.373, *p* = 0.010) and dietary phenylalanine intake (r = 0.361, *p* = 0.008) (Table 7).

A multivariable linear regression analysis revealed a statistically significant model (R^2^ = 0.274, adjusted R^2^ = 0.224, F = 5.474, *p* < 0.001), accounting for 27.4% of the variance in plasma phenylalanine concentrations. After adjusting for age, gender, BMI, nutritional intake, protein equivalent intake from Phe-free protein substitutes, and physical activity level, BMI (β = 0.410, *p* < 0.001) and male gender (β = 0.413, *p* = 0.001) were found to be independently correlated with plasma phenylalanine concentrations. Age, protein equivalent intake from Phe-free protein substitutes, physical activity level, and energy intake had no significant correlation with plasma phenylalanine concentrations. No significant multicollinearity was observed (all VIF values < 5) (Table 8).

## 4. Discussion

The prevalence of PKU varies worldwide. In Europe, the mean prevalence is approximately 1:10,000 newborns, and it is most commonly encountered in Ireland and Turkey. According to the statistics of the Turkish Ministry of Health, the disease prevalence is one in 5.500 births, which is believed to be caused partly by the high rate of consanguinity within the population [20,21]. According to the IDMP-PKU study conducted in three tertiary pediatric metabolic clinics in Türkiye, approximately 1 in 4 patients (26.5%) were adults [21]. Because of the decreased frequency of clinic visits and lack of experienced dietitians working with adult PKU patients, this study aimed to evaluate the nutritional status together with physical activity levels of adult PKU patients in Türkiye.

While lifelong dietary treatment forms the core of the disease’s management, it is known that adherence to the treatment decreases with age [22]. It is often observed that phenylalanine levels in phenylketonuria patients during adolescence and adulthood are outside the target ranges, indicating insufficient adherence to the diet [23,24]. In a study conducted on adolescent and adult phenylketonuria patients in England and Austria, it was shown that 80% of them had blood phenylalanine levels above the target limits due to non-compliance [24]. In the USA, 77% of them failed to adhere to the diet [25]. Pinto A et al. found that in 442 adult PKU patients from nine European centers between 2012 and 2018, the percentage of Phe levels within the target range declined with increasing age (from 64% to 40% aged 19–30 yrs and ≥41 yrs, respectively) [26]. In our study the percentage of Phe levels within the target range is higher in females (43.4%) compared to males (25.5%). While these results support previous evidence of metabolic control in adult PKU patients, the relatively higher target-range achievement observed in females may indicate better metabolic control in our patients. Poor dietary adherence and elevated blood phenylalanine levels in adult patients can be attributed to their failure to consume protein substitutes (25). In this study, 4% of the patients (1 male, 3 females) reported that they did not follow the diet at all and did not consume the amino acid mixture, and thirty-four patients (16 males, 18 females) took a protein substitute once or twice a day. Patients reported avoiding protein substitutes due to poor palatability and perceived social stigmatization in social contexts. The similar number of patients with suboptimal adherence to amino acid mixture in both groups may indicate that differences in dietary phenylalanine intake (1218.9 ± 612.5 mg/day in males, 936.2 ± 683.4 mg/day in females) could contribute to variations in blood Phe concentrations (972 ± 462 µmol/L in males, 714 ± 372 µmol/L in females) in this adult cohort; however, this observation should be interpreted cautiously.

Weight gain has been identified as a comorbidity associated with phenylketonuria recently [27]. This study revealed that 55% of the patients were overweight or obese (59.6% in males, 51% in females). This prevalence appears comparable to that reported in the general Turkish population. In Türkiye, the overall prevalence of overweight and obesity has been reported to be approximately 56% (the prevalence of obesity and overweight in 2022 was 16.8% and 40.4% in males and 23.6% and 30.9% in females, respectively) [28]. However, differences in study populations and methodologies should be considered when interpreting this comparison. The prevalence of overweight and obesity in our study population was comparable to that reported in the general Turkish population; however, dietary treatment and metabolic management in PKU differ substantially from those of the general population. Therefore, the management of excess body weight remains clinically important in PKU, as it may contribute to improved treatment adherence and better long-term health outcomes. Although overweight and obesity were common in this adult PKU cohort, the present study was not designed to identify causal mechanisms underlying these findings. Since dietary intake was assessed using a singular 24 h dietary recall and the lack of evaluation of detailed nutritional characteristics such as dietary fiber, glycemic index, glycemic load, food processing level, and quality of carbohydrate sources, no conclusions can be made regarding the impact of specific dietary components on body weight status. Therefore, these findings should be interpreted as descriptive observations rather than indications of a causal relationship.

To prevent obesity, it is recommended that exercise and sufficient physical activity be incorporated together into the individual’s life in this patient group [29]. Although adult phenylketonuria patients have a high tendency towards obesity, ideal physical development is an achievable goal independent of dietary restrictions [30]. Robertson et al. [31] have shown that where body mass index increased with the patients’ age, it was emphasized that there was no longitudinal data showing whether this situation was due to excessive energy intake or insufficient physical activity as age progressed.

In a phenylalanine-restricted diet, natural protein intake is reduced, and dietary patterns may differ from those of the general population due to treatment-related dietary restrictions. In terms of energy, carbohydrate, protein, and fat intakes, there was no significant difference between blood Phe classification groups in both genders. Only in males was it found that the carbohydrate intake with blood phenylalanine levels ≤ 600 µmol/L was higher than those with >600 µmol/L (*p* < 0.05). This finding may reflect differences in dietary adherence patterns; however, given the cross-sectional design of the study, these results should be interpreted cautiously.

The effects of physical activity on health go beyond just health [30]. Therefore, it is necessary to ensure the adoption and implementation of global guidelines for a healthier and more active future worldwide [30]. Exercise is one of the most important components of a healthy lifestyle, and individuals with phenylketonuria should be encouraged to engage in regular physical activity as outlined in the World Health Organization’s physical activity guidelines published in 2020 [30]. The World Health Organization recommends that adults engage in 150–300 min of moderate-intensity or 75–150 min of high-intensity physical activity per week, or an equivalent combination of moderate- and high-intensity aerobic physical activity. At the same time, the guidelines recommend regular muscle-strengthening activities for all age groups [30]. The American Physical Activity Guidelines also emphasize that similar amounts of physical activity have positive effects on improving and enhancing health [32]. A guide for phenylketonuria patients who exercise or play sports has not been published [33]. When creating a suitable nutrition plan for these patients, there are certain restrictions, such as limiting phenylalanine intake while ensuring adequate energy and nutrient supply to support their physical activity [33]. In patients who exercise, it is necessary to carefully plan nutrition to support exercise without affecting blood phenylalanine levels [34]. This study found that adult male patients with PKU who were categorized as sufficiently active showed significantly higher of energy intake, Phe-free protein substitute intake, and BMI values than their less active counterparts. Nevertheless, due to the unavailability of body composition metrics such as fat mass, lean mass, waist circumference, bioimpedance analysis, or DXA measures, BMI alone is insufficient to differentiate adiposity from lean body mass. Therefore, the physiological significance of these findings remains uncertain and should be interpreted cautiously. In women, sufficiently active individuals tended to have lower plasma Phe concentrations and higher BMI values, but these differences did not reach statistical significance and should therefore be interpreted with caution. The lack of statistical significance may partly be related to the relatively small number of women in the sufficiently active group. Previous studies in adult PKU patients who performed acute aerobic exercise reported that the activity did not affect the patients’ blood Phe levels, but patients with inadequate metabolic control had difficulty maintaining their oxygen consumption levels during exercise [35].

The main goals of the diet are a high-carbohydrate diet, careful monitoring of hydration status, and the timing of amino acid mixture intake during the post-exercise recovery process [33]. Although there is an inverse relationship between physical activity and obesity, there is no study conducted in this area at PKU. This study revealed that 59.6% of the males and 51% of the females were overweight and obese. Although males reported greater energy expenditure and higher energy intake than females, these findings do not allow conclusions regarding the mechanisms underlying gender-related differences in anthropometric or metabolic outcomes. These observations should therefore be interpreted cautiously.

## 5. Limitations

This study has some limitations. First, the cross-sectional design precludes causal inference; therefore, longitudinal studies are needed to confirm these associations. Secondly, although this single-center study included a relatively valuable sample of adults with PKU, the findings may not be fully generalizable to broader populations because the study was conducted in a single center in Türkiye, where cultural, dietary, and healthcare-related characteristics may differ from those of other populations. In addition, the 24 h retrospective food consumption record does not reflect the long term and can be affected by a momentary situation (such as travel, etc.), so long-term monitoring (including at least three weekdays and weekends) could have strengthened our results. Dietary intake was assessed using a single 24 h dietary recall; therefore, habitual intake patterns may not have been fully captured. Furthermore, body composition was not directly evaluated; thus, BMI alone may not precisely represent adiposity or the distribution of lean body mass. Although IPAQ is a validated instrument, self-reported physical activity assessment may be affected by recall bias and overestimation. For the evaluation of physical activity level, more effective assessment methods, such as pedometers, could be used.

These limitations should be considered when interpreting the findings. Future studies should address these issues by employing adequately powered sample sizes and larger multicenter cohorts and longitudinal studies, while also incorporating appropriate dietary considerations and addressing methodological shortcomings.

## 6. Conclusions

In phenylketonuria (PKU), lifelong dietary treatment remains the cornerstone of disease management; however, adherence is frequently suboptimal, particularly in adulthood, which may adversely affect nutritional status and metabolic control.

There are still limited studies in the literature that evaluate nutritional intake, anthropometric characteristics and physical activity levels in adult PKU patients and examine their effect on metabolic control parameters; therefore, this study was conducted to help address this gap. The present study revealed that adults with PKU exhibited a significant prevalence of overweight and obesity, alongside variations in nutritional intake and physical activity behavior. Physical activity levels correlated with many dietary and metabolic variables; however, due to the cross-sectional design, causal relationships cannot be determined. These findings underscore the significance of consistent nutritional evaluation and surveillance of lifestyle-related variables in adults with PKU. Further longitudinal studies are required to clarify the links among physical activity, nutritional intake and metabolic regulation.

## Figures and Tables

**Table 1:** Plasma Phenylalanine Concentrations and Age at Diagnosis of the Patients by Gender.

Variable	Males (*n* = 47)		Females (*n* = 53)		Total (*n* = 100)	
	Mean ± SD	Median-IQR	Mean ± SD	Median-IQR	Mean ± SD	Median-IQR
Newborn plasma Phe concentration (µmol/L)	1662 ± 412	1626 (1268–2070)	1728 ± 558	1728 (1212–2058)	1698 ± 582	1710 (1240–2040)
Age at diagnosis (days)	55.8 ± 87.5	30.0 (15.0–50.0)	46.3 ± 52.8	30.0 (14.0–60.0)	50.8 ± 71.0	30.0 (14.0–58.5)
Last measured plasma Phe concentration (µmol/L)	972 ± 462	870 (594–1332)	714 ± 372	636 (432–990)	834 ± 438	774 (516–1122)
**Classification according to the day of diagnosis**	**Males** ***n* (%)**		**Females** ***n* (%)**		**Total** ***n* (%)**	
Early diagnosis < 3 months	42 (89.4)		48 (90.6)		90 (90.0)	
Late diagnosis ≥ 3 months	5 (10.6)		5 (9.4)		10 (10.0)	
**Classification according to the median plasma Phe concentration**						
≤600 µmol/L	12 (25.5)		23 (43.4)		35 (35.0)	
>600 µmol/L	35 (74.5)		30 (56.6)		65 (65.0)	

SD, standard deviation; IQR, interquartile range; Phe, phenylalanine; *n*, number of participants.

**Table 2:** Anthropometric Measurements of Patients with Phenylketonuria.

Variable	Males (*n* = 47)		Females (*n* = 53)		Total (*n* = 100)	
	Mean ± SD	Median-IQR	Mean ± SD	Median-IQR	Mean ± SD	Median-IQR
Weight (kg)	77.0 ± 14.9	73.0 (66.2–85.5)	63.1 ± 15.7	62.5 (51.5–69.9)	69.6 ± 16.8	68.1 (57.7–81.1)
Height (cm)	172.2 ± 6.0	173.0 (169.0–175.7)	156.1 ± 13.9	157.5 (151.5–162.0)	163.6 ± 13.5	164.8 (156.5–172.9)
BMI (kg/m^2^)	25.9 ± 4.5	26.1 (22.3–28.4)	27.3 ± 5.3	25.2 (22.6–27.3)	26.6 ± 4.9	25.5 (22.5–28.2)
**BMI Classification**	**Males** ***n* (%)**		**Females** ***n* (%)**		**Total** ***n* (%)**	
Underweight (BMI < 18.50)	-		5 (9.4)		5 (5.0)	
Normal (18.50 ≤ BMI < 25)	19 (40.4)		21 (39.6)		40 (40.0)	
Overweight (25 ≤ BMI < 30)	22 (46.8)		17 (32.1)		39 (39.0)	
Obese (BMI ≥ 30)	6 (12.8)		10 (18.9)		16 (16.0)	
**Total**	47 (100.0)		53 (100.0)		100 (100.0)	

BMI, body mass index; SD, standard deviation; IQR, interquartile range; *n*, number of participants.

**Table 3:** Energy and Nutrient Intakes in PKU Patients by Gender and Blood Phenylalanine Classification.

Energy and Nutrient Intakes	Total		Blood Phe Level ≤ 600 µmol/L		Blood Phe Level > 600 µmol/L		p1	p2
	Males (*n* = 47) Mean ± SD (Min–Max)	Females (*n* = 53) Mean ± SD (Min–Max)	Males (*n* = 12) Mean ± SD (Min–Max)	Females (*n* = 23) Mean ± SD (Min–Max)	Males (*n* = 35) Mean ± SD (Min–Max)	Females (*n* = 30) Mean ± SD (Min–Max)		
Energy intake ^†^ (kcal/day)	2443.8 ± 384.6 (1727.2–3714.6)	1822.5 ± 312.7 (1294.9–2980.4)	2564.8 ± 428.1 (1912.8–3714.6)	1853.3 ± 344.8 (1359.5–2980.4)	2381.3 ± 351.1 (1727.2–3310.5)	1782.3 ± 267.3 (1294.9–2297.8)	0.125	0.441
Carbohydrate ^†^ (g/day)	362.1 ± 83.6 (231.5–612.9)	266.1 ± 56.2 (168.3–459.3)	398.6 ± 96.5 ^a^ (231.5–611.4)	271.5 ± 62.6 (168.3–459.3)	343.2 ± 70.5 ^b^ (239.6–612.9)	259.0 ± 46.9 (168.4–342.6)	0.032	0.471
Carbohydrate (%TE)	60.9 ± 7.4 (50.0–78.0)	60.5 ± 6.6 (46.0–74.0)	64.1 ± 8.9 ^a^ (50–78)	61.0 ± 7.0 (46.0–74.0)	59.3 ± 6 ^b^ (51.0–77.0)	59.7 ± 6.0 (50.0–74.0)	0.031	0.482
Fat (g/day)	72.5 ± 22.1 (35.5–111.9)	53.5 ± 16.2 (18.5–85.2)	68.3 ± 24.5 (35.5–111.9)	54.0 ± 17.7 (18.5–84.3)	74.6 ± 20.8 (38.9–111.5)	52.8 ± 14.3 (18.6–85.2)	0.362	0.796
Fat (%TE)	26.8 ± 7.4 (12.0–40.0)	26.4 ± 7.2 (12.0–42.0)	24.3 ± 8.4 (12.0–39.0)	26.3 ± 7.6 (12.0–39.0)	28.1 ± 6.6 (13.0–40.0)	26.5 ± 6.7 (13.0–42.0)	0.097	0.929
Protein ^†^ (g/day)	71.7 ± 14.6 (45.1–114.9)	56.6 ± 14.2 (33.8–116.2)	70.7 ± 10.4 (54.3–87.7)	54.9 ± 14.7 (34.8–116.2)	72.1 ± 16.5 (45.1–114.9)	58.8 ± 13.4 (33.8–80.9)	0.929	0.315
Protein ^†^ (%TE)	12.2 ± 2.6 (8.0–22.0)	13.0 ± 3.2 (8.0–21.0)	11.6 ± 1.6 (8.0–15.0)	12.7 ± 3.4 (8.0–21.0)	12.6 ± 2.9 (9.0–22.0)	13.5 ± 2.9 (8.0–19.0)	0.240	0.284
Protein ^†^ (g/kg)	1.0 ± 0.4 (0.5–1.7)	0.9 ± 0.2 (0.4–1.5)	1.0 ± 0.2 (0.7–1.5)	1.0 ± 0.2 (0.5–1.3)	0.9 ± 0.3 (0.5–1.7)	0.9 ± 0.3 (0.4–1.5)	0.309	0.108
Protein equivalent from Phe-free protein substitutes ^†^ (g/day) (*n* = 96)	44.7 ± 10.0 (20.0–70.0)	40.0 ± 14.3 (14.0–105.0)	46.7 ± 8.9 (33.0–60.0)	40.6 ± 17.5 (14.0–105.0)	43.8 ± 10.5 (20.0–70.0)	39.3 ± 8.9 (20.0–60.0)	0.310	0.899
Protein equivalent from Phe-free protein substitutes (% of total protein intake)	62.9 ± 9.6 (42.1–80.0)	69.6 ± 13.1 (24.2–90.4)	66.7 ± 9.3 (44.5–77.9)	71.9 ± 14.5 (24.2–90.4)	61.1 ± 9.4 (42.1–80.0)	66.6 ± 10.6 (49.5–90.0)	0.064	0.165
Phenylalanine ^†^ (mg/day)	1218.9 ± 612.5 (456.0–3710.9)	936.2 ± 683.4 (255.1–3143.2)	1150.7 ± 776.9 (478.8–3710.9)	829.9 ± 611.4 (285.6–2669.2)	1254.1 ± 519.3 (456.0–2642.2)	1074.9 ± 758.8 (255.1–3143.2)	0.250	0.208

(1) Independent samples *t*-test, *p* < 0.05 (p1 refers to males and p2 refers to females.) (2) ^†^ Values were analyzed after logarithmic transformation when normality assumptions were not met. (3) SD, standard deviation; IQR, interquartile range; Phe, phenylalanine; %TE, percentage of total energy intake. (4) Protein equivalent contribution from Phe-free protein substitutes was calculated as the proportion of total daily protein intake derived from protein substitutes. (5) Different superscript letters (a, b) indicate statistically significant differences between groups within the same row (*p* < 0.05).

**Table 4:** Physical Activity Parameters and PAL Classification of the Patients by Gender.

Physical Activity Level (MET-min/Week)	Males (*n* = 47)	Females (*n* = 53)	Total (*n* = 100)
	Median-IQR	Median-IQR	Median-IQR
Total Physical Activity	3576 (1848–5820)	1230 (396–1980)	1860 (900–3657)
Vigorous Physical Activity	960 (0–2880) (*n* = 34)	0 (0–0) (*n* = 11)	0 (0–1200) (*n* = 45)
Moderate Total Physical Activity	800 (480–1440) (*n* = 43)	0 (0–480) (*n* = 24)	480 (0–960) (*n* = 67)
Walking Physical Activity	1386 (792–2376) (*n* = 47)	792 (396–1386) (*n* = 53)	990 (561–1980) (*n* = 100)
Sitting Time (min)	420 (240–480) (*n* = 47)	420 (180–480) (*n* = 53)	420 (190–480) (*n* = 100)
**Classification of PAL**	***n* (%)**	***n* (%)**	***n* (%)**
Inactive	1 (2.1)	16 (30.2)	17 (17.0)
Minimally Active	13 (27.7)	27 (50.9)	40 (40.0)
Sufficiently Active	33 (70.2)	10 (18.9)	43 (43.0)

IPAQ, International Physical Activity Questionnaire; PAL, physical activity level; MET, metabolic equivalent of task; IQR, interquartile range; min, minutes; *n*, number of participants.

**Table 5:** Clinical, Anthropometric, and Dietary Characteristics of Adult PKU Patients by Gender and Physical Activity Level.

	Total		Low-Active		Sufficiently Active		p1	p2
	Males (*n* = 47) Mean ± SD (Min–Max)	Females (*n* = 53) Mean ± SD (Min–Max)	Males (*n* = 14) Mean ± SD (Min–Max)	Females (*n* = 43) Mean ± SD (Min–Max)	Males (*n* = 33) Mean ± SD (Min–Max)	Females (*n* = 10) Mean ± SD (Min–Max)		
Energy intake (kcal/day)	2443.8 ± 384.6 (1727.2–3714.6)	1822.5 ± 312.7 (1294.9–2980.4)	2158 ± 201.3 (1727.2–2444.9)	1782 ± 267.2 (1294.9–2658.4)	2565 ± 381.1 (1961.3–3714.6)	1996.6 ± 436.1 (1467.5–2980.4)	<0.0001	0.067
Protein (g/day)	71.7 ± 14.6 (45.1–114.9)	56.6 ± 14.2 (33.8–116.2)	66.2 ± 12.6 (45.1–94.9)	56.1 ± 12 (34.8–80.9)	74 ± 15 (50.8–114.9)	58.9 ± 22.1 (33.8–116.2)	0.092	0.787
Protein equivalent from Phe-free protein substitute (g/day) (*n* = 96)	44.7 ± 10 (20–70)	40 ± 14.3 (14–105)	39.1 ± 8.5 (20–50)	39.1 ± 10.8 (14–75)	47.2 ± 9.7 (30–70)	44.6 ± 26.3 (20–105)	0.008	0.672
Phenylalanine (mg/day)	1218.9 ± 612.5 (456–3710.9)	936.2 ± 683.4 (255.1–3143.2)	1194.1 ± 491.5 (652.1–2454.9)	920.6 ± 626.2 (255.1–3143.2)	1229.4 ± 663.9 (456–3710.9)	1003.2 ± 928.9 (307–2813.6)	0.910	0.918
Last measured plasma Phe concentration (µmol/L)	972 ± 462 (300–2286)	714 ± 372 (48–1446)	1002 ± 432 (318–1998)	732 ± 384 (48–1446)	960 ± 480 (300–2286)	618 ± 336 (126–1188)	0.756	0.394
BMI (kg/m^2^)	25.9 ± 4.5 (19–40.7)	25.1 ± 5.3 (15.6–43)	23.7 ± 4.1 (19–31.4)	24.8 ± 5.3 (15.6–43)	26.9 ± 4.4 (19–40.7)	26.6 ± 5.5 (19.3–36.3)	0.025	0.341

(1) Low-active includes inactive and minimally active participants. (2)Independent samples *t*-test, *p* < 0.05 (p1 refers to comparisons between male physical activity groups; p2 refers to comparisons between females). (3)Values were analyzed after logarithmic transformation when normality assumptions were not met. (4) BMI, body mass index; Phe, phenylalanine; SD, standard deviation; min, minimum; max, maximum; *n*, number of participants.

**Table 6:** BMI Classification According to Physical Activity Levels in Adult Patients with PKU.

BMI Classification	Classification of IPAQ			
	Total *n* (%)	Inactive *n* (%)	Minimally Active *n* (%)	Sufficiently Active *n* (%)
Underweight (BMI < 18.50)	5 (100.0)	3 (60.0)	2 (40.0)	-
Normal (18.50 ≤ BMI < 25)	40 (100.0)	5 (12.5)	21 (52.5)	14 (35.0)
Overweight (25 ≤ BMI < 30)	39 (100.0)	7 (17.9)	10 (25.6)	22 (56.4)
Obese (BMI ≥ 30)	16 (100.0)	2 (12.5)	7 (43.8)	7 (43.8)
**Total**	100 (100.0)	17 (17.0)	40 (40.0)	43 (43.0)

BMI, body mass index; IPAQ, International Physical Activity Questionnaire; *n*, number of participants.

**Table 7:** Spearman Correlation Analysis of Physical Activity, Anthropometric Parameters, Plasma Phenylalanine Concentrations, and Dietary Variables in Adult Patients with PKU.

Variables	IPAQ Score (MET-min/Week)	BMI (kg/m^2^)	Last Measured Plasma Phe Level (µmol/L)	Energy Intake (kcal/Day)	Protein (g/Day)	Phenylalanine (mg/Day)	Protein Equivalent from Phe-Free Protein Substitutes (g/Day) (*n* = 96)
IPAQ score (MET-min/week)	-	0.087 (*p* = 0.535)	−0.064 (*p* = 0.650)	0.107 (*p* = 0.446)	0.199 (*p* = 0.153)	0.043 (*p* = 0.759)	0.142 (*p* = 0.336)
BMI (kg/m^2^)	0.186 (*p* = 0.211)	-	0.276 * **(*p* = 0.045)**	0.257 (*p* = 0.063)	0.338 * **(*p* = 0.013)**	0.104 (*p* = 0.458)	0.292 * **(*p* = 0.044)**
Last measured plasma Phe level (µmol/L)	−0.018 (*p* = 0.906)	0.434 ** **(*p* = 0.002)**	-	−0.018 (*p* = 0.897)	0.216 (*p* = 0.121)	0.283 * **(*p* = 0.040)**	0.006 (*p* = 0.969)
Energy intake (kcal/day)	0.443 ** **(*p* = 0.002)**	0.214 (*p* = 0.149)	−0.153 (*p* = 0.306)	-	0.269 (*p* = 0.052)	0.137 (*p* = 0.329)	0.141 (*p* = 0.338)
Protein (g/day)	0.108 (*p* = 0.470)	0.215 (*p* = 0.146)	0.057 (*p* = 0.702)	0.373 ** **(*p* = 0.010)**	-	0.361 ** **(*p* = 0.008)**	0.776 ** **(<*p* = 0.001)**
Phenylalanine (mg/day)	0.092 (*p* = 0.540)	0.085 (*p* = 0.569)	0.206 (*p* = 0.164)	0.206 (*p* = 0.164)	0.538 ** **(<*p* = 0.001)**	-	−0.007 (*p* = 0.963)
Protein equivalent from Phe-free protein substitutes (g/day) (*n* = 96)	0.092 (*p* = 0.543)	0.196 (*p* = 0.191)	−0.071 (*p* = 0.638)	0.187 (*p* = 0.214)	0.657 ** **(<*p* = 0.001)**	−0.170 (*p* = 0.259)	-

Values are presented as Spearman correlation coefficients (r) with corresponding *p*-values in parentheses. The lower-left triangle represents data for male participants, whereas the upper-right triangle represents data for female participants. Statistically significant correlations are indicated as * *p* < 0.05 and ** *p* < 0.01. IPAQ, International Physical Activity Questionnaire; BMI, body mass index; Phe, phenylalanine. Bold values indicate statistically significant results (*p* < 0.05).

**Table 8:** Multivariable Linear Regression Analysis of Factors Associated with Plasma Phenylalanine Concentrations in Adults Patients with PKU.

	Unstandardized Coefficients	95.0% Confidence Interval for β		Standardized Coefficients	*p*-Value
	β	Lower Bound	Upper Bound	Beta	
(Constant)	40,979	−631,595	713,552		0.904
BMI (kg/m^2^)	38,098	19,602	56,595	0.410	**<0.001**
Gender	355,082	141,222	568,941	0.413	**0.001**
Age (years)	11,330	−4092	26,753	0.141	0.148
Protein equivalent from Phe-free protein substitutes (g/day)	−4768	−11,946	2410	−0.138	0.190
IPAQ score (MET-min/week)	0.006	−0.023	0.035	0.049	0.691
Energy intake (kcal/day)	−0.200	−0.488	0.088	−0.220	0.170

Model adjusted for age, gender, BMI, energy intake, protein substitute intake, and physical activity level. Model fit: R^2^ = 0.274, adjusted R^2^ = 0.224, F = 5.474, *p* << 0.001. Bold values indicate statistically significant results (*p* < 0.05).

## Data Availability

The original contributions presented in the study are included in the article. Additionally, for further information, the corresponding author can be contacted.

## Abbreviations

In this article, the following abbreviations have been used:

AAM	Amino Acid Mixture
ACMG	American College of Medical Genetics and Genomics
BMI	Body Mass Index
BEBIS	Nutrition Information System (Beslenme Bilgi Sistemi)
cPKU	Classical Phenylketonuria
HPLC	High-Performance Liquid Chromatography
IDMP-PKU	Integrated Disease Management Program for Phenylketonuria
IPAQ	International Physical Activity Questionnaire
IQR	Interquartile Range
MET	Metabolic Equivalent of Task
PAL	Physical Activity Level
PAH	Phenylalanine Hydroxylase
Phe	Phenylalanine
PKU	Phenylketonuria
SD	Standard Deviation
TE	Total Energy
%TE	Percentage of Total Energy Intake
WHO	World Health Organization
